# Addiction, Passion, or Confusion? New Theoretical Insights on Exercise Addiction Research From the Case Study of a Female Body Builder

**DOI:** 10.5964/ejop.v14i2.1545

**Published:** 2018-06-19

**Authors:** Attila Szabo

**Affiliations:** a Institute of Health Promotion and Sport Sciences, ELTE Eötvös Loránd University, Budapest, Hungary; b Institute of Psychology, ELTE Eötvös Loránd University, Budapest, Hungary; Webster University Geneva, Geneva, Switzerland

**Keywords:** commitment, dependence, deprivation, mood, sport

## Abstract

Exercise addiction is widely studied in sport science and psychology, but at this time it is not recognized as an independently diagnosable mental or psychiatric disorder. Indeed, studies on exercise addiction assess a level of risk for disordered exercise behaviour, characterized by lack of control and negative personal consequences. It is argued that commitment and passion are two overlapping features of high exercise involvement which obscure the fine line between healthy and unhealthy exercise. The present case study examined a successful female body builder who initially claimed that she was addicted to exercise. During an interview she also completed three questionnaires and her appraisal of well-being in eight life domains were assessed at present, as well as retrospectively before her intensive involvement with exercise. She was screened under the Non-Substance Related Disorders category of Substance-Related and Addictive Disorders classification of DSM-5 for gambling, by replacing the word "gambling" with "exercise". Although she was susceptible to exercise addiction, attained high scores on obsessive passion, exhibited more than four symptoms on the DSM list, she exhibited no signs of loss of control and she mainly reported positive experiences associated with her exercise behaviour. She has obtained a nearly maximum score on commitment to exercise and high score on harmonious passion. Almost all aspects of her life have changed in positive direction after getting intensely involved in exercise. This case illustrates that the current scholastic path to the study of exercise addiction may be obscured by ambiguous assumptions and unilateral quantitative focus.

## The Dark Side of Exercise Behaviour

Physical activity embodies all forms of movements that require energy expenditure to sustain the work performed by the skeletal muscles; sports and exercise are its two subcategories (Caspersen, Powell, & Christenson, 1985). Exercise is considered as a health-oriented behaviour in the contemporary increasingly sedentary human lifestyle (Foulds, Bredin, Charlesworth, Ivey, & Warburton, 2014). However, there is a pathological aspect of exercise as well (Szabo, 2000). Exercising to the point where the person *loses control* over the pattern and the volume of exercise, which then becomes an obligatory, or a *must do* activity, results in negative physical and/or mental consequences. The emerging dysfunction is defined as *exercise addiction* (Griffiths, 1997; Szabo, 2010). Some authors also refer to the condition as *exercise dependence* (Hausenblas & Downs, 2002). However, dependence is incorrect, or rather incomplete, as Goodman (1990) noted earlier, since dependence is only one of the two aspects of addiction, the other being compulsion. To establish the presence of dependence, tolerance and withdrawal symptoms should be associated with the behaviour (Miele, Tilly, First, & Frances, 1990). Compulsion may be defined as a strong "egodystonic" (distinct from self) urge to engage in an activity to remove the anxiety triggered by the urge (Brewer & Potenza, 2008). Considering this definition, compulsion is a component of addiction that involves negative reinforcement. The latter refers to the elimination, alleviation, or avoidance of something unpleasant/undesired through which the rate of the successful/rewarding behaviour increases in the future (Doggett & Koegel, 2013). Further, dependence might have a positive outcome and it could be mediated by positive reinforcement, defined as the granting of a desired/pleasant stimulus, conditional upon a behaviour, that results in greater frequency of the behaviour in the future (Doggett & Koegel, 2013). While addiction may involve both positive reinforcement (a pleasant gain) and negative reinforcement (successful avoidance), the outcome in addiction is always negative (Goodman, 1990; Szabo, 2010). The obligatory nature of the exercise behaviour (i.e., it must be accomplished to avoid something noxious, like anxiety or withdrawal symptoms) reflects its primary regulation through negative reinforcement (Egorov & Szabo, 2013; Szabo, Griffiths, & Demetrovics, 2013b).

### Exercise Addiction

Exercise addiction is classified as a *behavioural addiction* (Egorov & Szabo, 2013) akin to gambling disorder, but due to insufficient empirical evidence for being a mental dysfunction, the condition is not listed in the latest (fifth) edition of the Diagnostic and Statistical Manual of Mental Disorders (DSM-5; American Psychiatric Association, 2013). To date, there are only a few published case studies in the literature that report *genuine cases* of pathological exercise addiction (Szabo, Griffiths, De La Vega, Mervó, & Demetrovics, 2015). Most of these reports were published over the past two decades (e.g., an early report by Veale, 1995, and a recent case by Hausenblas, Schreiber, & Smoliga, 2017). They usually present the symptoms and the aetiology of the dysfunctional behaviour accompanied by a theoretical analysis. What is common between these case reports is that in all instances the affected individual evidences exercise abuse and, consequently, experiences severe negative health and social ailments.

The first case study in exercise addiction was a presented by Veale (1995) on a 27-year female runner who apart from her exercise had no other interests in life. A more frequently cited case study, published later by Griffiths (1997), covered the morbid exercise pattern of a 25-year-old female martial artist. Seven years later the case of a 50-year-old cyclist man, who also had a history of psychopathology and sexual addiction, was brought into the attention of the scholastic community (Kotbagi, Muller, Romo, & Kern, 2014). Then in a recently published book about exercise addiction, Schreiber and Hausenblas (2015) present eight cases of exercise addiction, starting with the case of the first author, then presenting the story of five women and two men mainly from the patients’ subjective perspective to make readers aware of how exercise addiction feels like. Therefore, dysfunctional behaviour in which the affected person uses exercise for either escape from hardship, coping with stress, hiding from responsibilities or conformity, or for any other reason, clearly exists. Although not all exercise addicts will share their story, the few reported cases do not justify the high volume of research on this topic. Indeed, if one runs a title-based search in Google Scholar™ using the paired words "exercise addiction" and then again using "exercise dependence" (another term used for the dysfunction), will find over 500 records. Therefore, the better understanding of exercise addiction, and its psychopathological aspects, is warranted.

### A Gap in the Exercise Addiction Literature

A major dilemma in the current literature is whether exercise addiction occurs only among recreational exercisers or professional athletes as well. Since behavioural addictions, in general, reflect an escape from a psychological problem (Griffiths, 1996), some authors argue that athletes are unlikely to escape into an activity that dominates their daily lives (Szabo et al., 2015,). Also, the characteristics of exercise addiction in light of the interactional model (Egorov & Szabo, 2013) distinguishes therapeutically oriented exercise from that performed for mastery reasons. Finally, the unusually high scores reported by athletes on various exercise addiction scales prompted scholars to suspect that high athletic commitment may be confounded with exercise addiction (Szabo et al., 2015). One approach to elucidate the problem is to use interviews with athletes thinking or claiming to be addicted to exercise and/or scoring high on exercise addiction measures (Szabo et al., 2015). This method could answer the question whether the interviewed (suspect to the disorder) athlete exhibits a dysfunction associated with specific patterns and high volumes of exercise or simply misinterprets the notion of addiction in relation to sport.

### Prevalence of the Risk for Exercise Addiction

The prevalence of the *risk* for exercise addiction is relatively rare, ranging from 0.3% to 0.5% as revealed in a representative sample from the general population (Mónok et al., 2012). However, in specific samples higher rates were found. In five studies with university students, Hausenblas and Downs (2002) reported prevalence rates ranging between 3.4% and 13.4%. Griffiths, Szabo, and Terry (2005) fund that 3.0% of university students were at risk of exercise addiction, but in a later work the rate emerged to be higher (6.9%) among sport science students (Szabo & Griffiths, 2007). Further, another study that surveyed 95 ultra-marathoners found only three people (3.2%) at-risk for exercise addiction (Allegre, Therme, & Griffiths, 2007). However, higher figures were reported for university athletes (7%–10%) and even higher for ultra-marathon runners (17%; Szabo, De La Vega, Ruiz-Barquín, & Rivera, 2013a). It should be emphasized that the reported figures were derived from questionnaires which only measure the presumed *risk* for exercise addiction and, therefore, cannot be considered as diagnostic tools (Szabo, 2010).

## Passion in Sport and Exercise

Heavy athletic involvement in sport is associated with passion (Vallerand et al., 2006). Passion seems to explain the exaggerated time devoted to sports or exercise, which is a common feature of athletic training and exercise addiction. Recently, based on a strong link established between exercise addiction and passion, it was suggested that "*The concept of exercise addiction is not a plain and independent construct and may not reflect a psychological dysfunction in the athletic population*" (De La Vega, Parastatidou, Ruíz-Barquín, & Szabo, 2016, p. 325). There is a dual model for passion comprised by obsessive and harmonious passion (Vallerand et al., 2003). As we have described in a recent study (Kovacsik et al., 2018), obsessive passion surfaces when the individual internalizes an activity in a controlled way and involvement is rigidly controlled; it is positively related to negative affect (Stenseng et al., 2011; Vallerand et al., 2003; Vallerand et al., 2007; Vallerand & Miquelon, 2007). The obsessively passionate person attaches great importance to activity contingencies, such as self-esteem and escape from problems which makes it hard for them to stop the passionate activity (Vallerand, 2010). Harmonious passion emerges when an activity is internalized autonomously and the individual engages in the activity with flexibility; it is associated with positive affect and it is inversely related to negative affect (Stenseng, Rise, & Kraft, 2011; Vallerand et al., 2003; Vallerand et al., 2006; Vallerand & Miquelon, 2007).

### Exercise Addiction and Passion: New Research Findings

Few empirical studies have examined the relationship between exercise addiction and passion. Obsessive passion was positively associated with exercise addiction in endurance sports as well as other leisure physical activities (Schipfer & Stoll, 2015; Stenseng et al., 2011). It was shown that obsessive passion is associated with all the dimensions of exercise addiction, including time, reduction in other activities, tolerance, withdrawal, continuance, intention effects, and absence of control. This link was not demonstrated for harmonious passion which was only related to time and tolerance (Paradis, Cooke, Martin, & Hall, 2013). Further, it was shown that harmoniously passionate exercisers can increase their exercise volume without neglecting life responsibilities. This is not the case for the obsessively passionate exercisers who spend exaggerated amounts of time on exercise while neglecting important responsibilities (Paradis et al., 2013). These findings are supported by a Greek research showing that obsessive passion has a stronger link to exercise addiction than harmonious passion (Parastatidou, Doganis, Theodorakis, & Vlachopoulos, 2014), and a Swedish study demonstrating that those at risk for exercise addiction also score higher on obsessive passion than those who are not at risk (Back, 2016).

The link between exercise addiction and passion, as a function of the athletic level of competition, was examined in a recent work studying a large sample of low- and high-level competitive athletes, and non-competitive leisure exercisers (De La Vega et al., 2016). In line with Parastatidou et al. (2014), the findings revealed that, by accounting for 37% of the total variance, obsessive passion was a predictor of exercise addiction while harmonious passion was not. These findings may not be surprising in the view of the controlling and being controlled aspects of passion and addiction, nor considering negative and positive reinforcement that could be related to the emergence and persistence of harmonious and obsessive passion.

With the recent evidence showing that passion may influence the measures of exercise addiction, in a similar manner as commitment to exercise does, the re-evaluation of the approach to the study of exercise addiction may be necessary. It is accepted that the common denominator between the healthy skill-oriented athletic training and the unhealthy escape into exercise is the unusually great time devoted to workouts (Szabo et al., 2015). Therefore, it is important to understand how a competing athlete feels about her/his exercise behaviour when she presents signs of exercise addictions on the bases of the current conceptualizations of the dysfunction.

## Aim of the Current Study

The aim of the current work was to examine the presence or absence of the currently known components of exercise addiction, in a young successful athlete who identified herself as addicted to exercise. The study relied on a personal interview in which apart from the verbal answers the interviewee also completed some popular questionnaires assessing the risk for exercise addiction, passion for exercise, exercise deprivation symptoms, as well as commitment to exercise, not for quantitative purposes, but to complement the interview with her relative (i.e., high-low) scoring directions on these questionnaires to check the agreement between the two methods (i.e., if she claims high commitment to exercise, is that also reflected in a high score on the commitment to exercise scale?). In past works it was suggested that for getting the sharpest image of exercise addiction nomothetic data (i.e., questionnaire scores) should be followed up with interviews (Szabo et al., 2015). The two methods jointly could provide the most accurate picture about one’s feeling states by blending the conscious with the first impression-based semi-conscious aspects.

## Method

### The Research Method

As predicted by the interactional model, which states that personal predispositions, exercise experience, and life stress interact in the aetiology of exercise addiction (Egorov & Szabo, 2013), the studied dysfunction is highly idiosyncratic. Whether a person is affected by exercise addiction can only be established by using a personal interview that considers the antecedents, current situations, training habits, and subjective psychological outcomes (Szabo et al., 2015). Indeed, questionnaires used in nomothetic research could only establish a "level of risk", but cannot clearly identify those who are affected by exercise addiction. Nevertheless, the inclusion of the usually adopted nomothetic measures might yield additional information to the qualitative data and reflect the match or the mismatch between the two. Therefore, as part of the interview the participant was also asked to rate the commonly accepted measures of exercise addiction. Her well-being was evaluated based on Swarbrick's (2006) eight life domains.

### The Participant

After a campus-wide search for participants in an interview - listing the conditions for participation that included the typical symptoms of exercise addiction (Griffiths, 2005) and high volume of exercise training - one female participant came forward and volunteered for the study. Her motive for volunteering was the expression of interest in better understanding her affinity for exercise and her exercise behaviour that (she claimed) has changed her life. The current case study was conducted with the ethical permission of the Research Ethics Committee in the Faculty of Education and Psychology at ELTE Eötvös Loránd University in Budapest. Prior to the study, the participant provided written consent for taking part in this work. Her fictive name in this work is Evelyn. At the time of the interview, she was a 24-year-old university graduated woman who lived in Budapest, Hungary. She worked in a managerial-assistant position, which was important to her. During her university studies she attended a gym relatively sporadically, but gradually she got increasingly involved in body building. After experiencing subjective and objective results, less than a year before the current interview, she has started to compete under the guidance of a coach and took part in two national and a major international competition. Thus, a shift from *exercise* to *sport,* or from an *exerciser-* to the *athletic status*, took place within the year of the current interview. Her early athletic results were very good considering her brief sporting history; she may be regarded as a highly successful athlete who has achieved three remarkable competition results within six months. She trained at least five times a week, for at least one and a half hour each time. Occasionally, Evelyn felt like working out in addition to her scheduled training. The reason for the extra workouts was guilt for not having enough training or missing a training session, as well as lack of personal satisfaction with the planned progress or the self-set goals. She received notable support, but also criticism, from friends on social media in context of her sport and her athletic physical appearance. This change in her appearance, however, was perceived positively by Evelyn. She had little time for socialization due to her work and training. The lack of a fulfilling social life appeared to be the greatest concern at the time of the study.

### Instruments: Measurements Complementing the Interview

It should be emphasized that this is not a nomothetic or quantitative study. It is a case study. Therefore, the tools used to complement the interview, while gathering numerical scores, should be still interpreted in a merely qualitative manner, such as *low, medium,* or *high* (like the needle of the odometer) within a given range. These tools were adopted to assess the concordance between interview data and the relative (i.e. high-low) scoring on the commonly used measures in exercise addiction research. For example, if the participant would claim that she is highly addicted to exercise, but would score on the lower end of the Exercise Addiction Inventory, her answers would be questionable.

The six-item Exercise Addiction Inventory (EAI; Terry, Szabo, & Griffiths, 2004) was used to assess her risk for exercise addiction. Items of the EAI include statements like: "Exercise is the most important thing in my life." or " If I have to miss an exercise session I feel moody and irritable.". These statements are rated on a 5-point Likert scale, ranging from 'strongly disagree' to 'strongly agree'. The maximum score is 30. A score of 24 or over suggest high risk for exercise addiction. The scales' internal reliability (Cronbach α) ranges between .61 to .80 in several cross-cultural samples (Griffiths et al., 2015; Mónok et al., 2012; Terry et al., 2004). The concurrent validity of the EAI, established through correlations with the Obligatory Exercise Questionnaire (Pasman & Thompson, 1988) and the Exercise Dependence Scale (Hausenblas & Downs, 2002), was .80 and .81, respectively. Its two-week test-retest reliability was .85 (Griffiths et al., 2005).

Passion was assessed using the revised Passion Scale (PS: Marsh et al., 2013). Its items include statements like: "My sport is in harmony with the other activities in my life." or "If I could, I would only do my sport.". This scale gauges harmonious- and obsessive passion across two 6-item subscales that are rated on a 7-point Likert scale, ranging from 'not agree at all' to 'very strongly agree'. The scales' internal reliability (Cronbach’s alpha) is .83 for harmonious passion and .86 for obsessive passion (Marsh et al., 2013). These values are higher in the exercise context, .88 for harmonious passion and .91 for obsessive passion (Parastatidou, Doganis, Theodorakis, & Vlachopoulos, 2012). The scale’s convergent validity was established through a series of statistically significant correlations with various passion determinants and passion outcomes (Parastatidou et al., 2012; Tables 2 and 3, pp. 129-130). The test-retest validity of the scale was established in a gambling context and it was .84 and .89 in two studies (Rousseau, Vallerand, Ratelle, Mageau, & Provencher, 2002). This scale also has a 5-item subscale that can be used for measuring a person's affinity for sport or any other activity. In the current study a validated Hungarian version of the scale was used with internal reliabilities of .80 and .88 (Tóth-Király, Bõthe, Rigó, & Orosz, 2017).

The 9-item Deprivation Sensations Scale (Robbins & Joseph, 1985) was used to measure nine symptom of exercise withdrawal, such as irritability or frustration, rated on a 7-point Likert Scale. The scales' internal reliability is .81 (Szabo, Frenkl, & Caputo, 1996). There are no reports about other psychometric properties of the scale. Finally, nine symptoms-based Non-Substance Related Disorders category of Substance-Related and Addictive Disorders classification of DSM-5 for gambling (American Psychiatric Association, 2013) was used by replacing 'gambling' with the word 'exercise'. These were built into the interview since the mere presence or absence of a symptom was the point, while the rest were presented in scalar-form using paper and pencil tools. (This is not a questionnaire, so no psychometric properties should be reported for the nine distinct categories, which was another reason for not presenting it as a paper and pencil instrument.)

### Procedure: The Interview

The interview took place in a quiet place without distraction and lasted approximately one hour. Evelyn completed the questionnaires and subsequently answered questions in the context of her training and emotional aspects of her sport. She then described and compared eight domains (Swarbrick, 2006) of her current well-being with the period preceding her intense involvement with body building. While, the author of this case study was (is) aware that retrospective answers could be biased, her subjective perception of the *change* (or difference) in the two situations was the most important. With Evelyn's consent the interview was recorded with a Smartphone's sound recorder application. The participant has approved the here published version of the interview.

## Results

### Quantitative Measures

**Figure 1:**
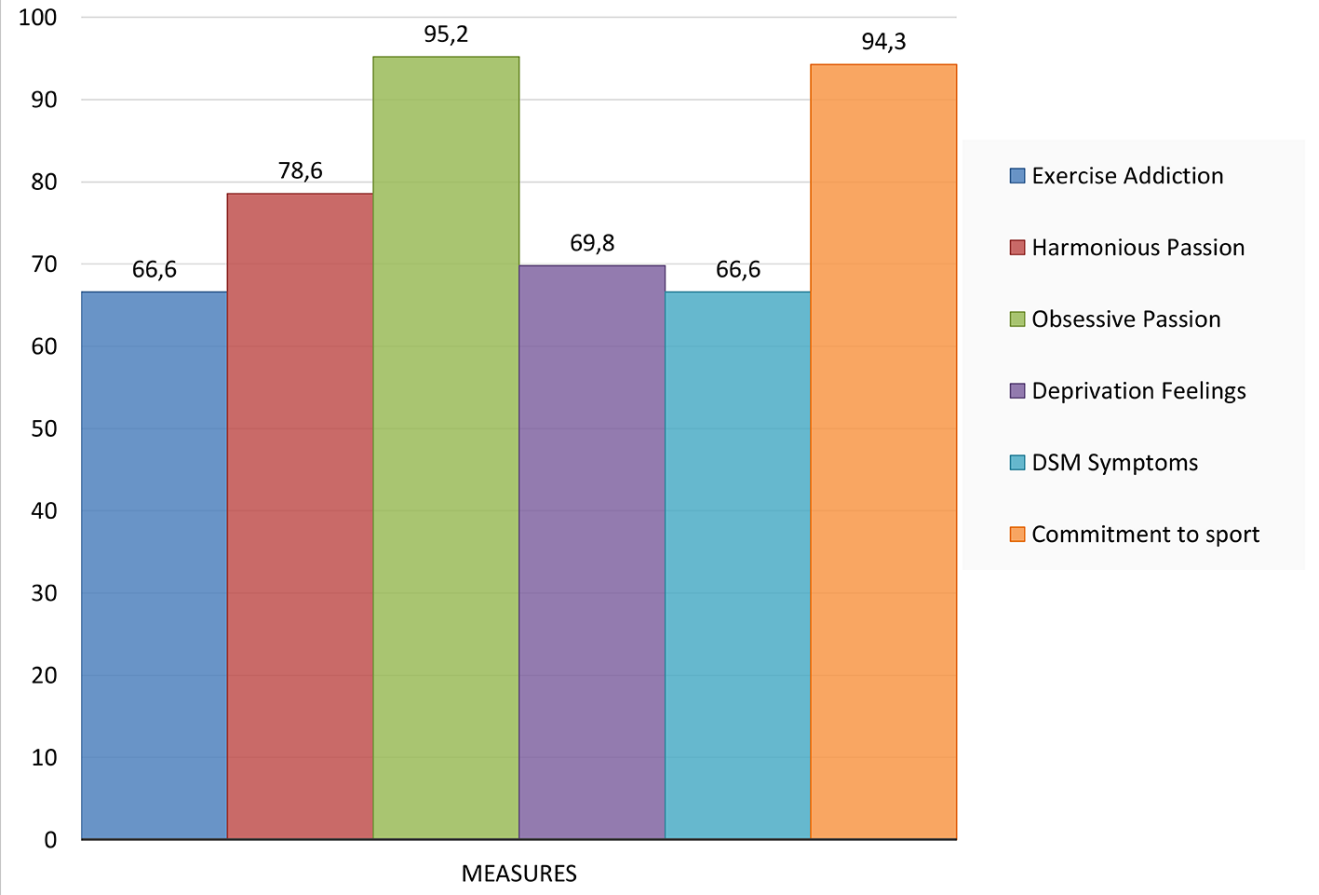
Evelyn's scores in percent (%) of the maximum (vertical axis range 0-100) on six measures of exercise behaviour.

On the EAI, Evelyn scored 20 out of 30 of the maximum score, which classifies her as 'symptomatic' based on the criteria of interpretation recommended by Terry et al. (2004), but not 'at risk' for exercise addiction for which the cut-off point is 24. Her mean EAI value was 3.33, which is like the mean score (3.15) of 384 regularly exercising university students (Sicilia, Alcaraz-Ibáñez, Lirola, & Burgueño, 2017) and 78 Spanish women athletes (mean score 3.04; Szabo et al., 2013a). On the Passion Scale she scored 33 out of the maximum of 42 on the harmonious passion, yielding a mean of 5.50, which is comparable, but slightly lower than the mean value (6.22) recently reported for exercising university students (Sicilia et al., 2017), however it matches closely the mean values (5.00, 5.56 and 5.84) reported in three studies with recreational and competitive athletes by Vallerand et al. (2006). Evelyn scored 40 out of 42 on obsessive passion. This score yields a mean value of 6.67 that is higher than the mean values (3.07, 3.84, and 4.34) reported by Vallerand et al. (2006), or the mean value (4.83) found in a recent Spanish work (Sicilia et al., 2017). Further, on the affinity or commitment items she scored 33 out of the possible 35 points, which obviously is near the maximum possible, so clearly it indicates a high score. On the Deprivation Sensation Scale Evelyn scored 44 out of 63 possible, which is higher than the values reported for exercisers in five different forms of exercise ranging from 19/63 in bowlers to 34/63 in aerobic exercisers, with a score of 30/63 (gender not reported) for eight body builders (Szabo et al., 1996). Her scores cannot be interpreted by considering the scores of other female body builders, because no such reports exist in the literature. Finally, she reported 6 out of 9 symptoms on the Non-Substance Related Disorders category of the DSM-5, that would classify her with disordered exercise behaviour (as an analogy to gambling disorder in the DSM-5). Her relative scores, in terms of percentages of the maximum (simply to illustrate the relative position [i.e., low, medium, high] of her answers), are summarized in Figure 1.

### Qualitative Measures

#### Training

Evelyn's training is scheduled. She trains in a team with 30 other women in which every person gets limited personal attention from the coach. She is not happy about that, but since the training routines are identical, Evelyn feels that she can manage her own training. The athletes are on a strict personalized diet designed, but not provided, by the coach. Evelyn does not take performance or growth stimulating agents. She is not keen about the strict diet that she must follow, in fact she considers it as the most inconvenient aspect of her training regimen. Otherwise, Evelyn shows no signs of any eating-related problems. She usually has two rest days a week, but sometimes - once biweekly - it happens that she feels the need to exercise in addition to her scheduled training. This happens when she misses a workout or does not attain a set goal in time. While she reports no major injuries due to her exercise, she has some backache that she manages with waist support and lower-weight use in some exercise routines. She claims that working out helps her in dealing with life stress. Evelyn believes that she has experienced some losses due to her intense involvement in exercise primarily in her social life and personal relationships, as well as in her study progress during the last term of her university studies. She has experienced conflicts at work due to exercise. Her major aspiration is to perform better in competitions and to get sponsors for her sport, so she can spend more time with the sport and have a more stable financial outlook. In fact, she would like to blend her sport into a career.

Matching the components model for addiction (Griffiths, 2005), Evelyn admits that her training represents the highest priority in her life. She is also aware of the distressing withdrawal symptoms if training is not possible for a reason. She often uses exercise for mood alteration, especially in dealing with stress. While, she thinks that she had no major interpersonal conflict because of her exercise, some conflict has occurred at her workplace. Two components that were rated low on the EAI could not be detected during the interview either. These were tolerance and relapse; she did not increase her amount of training in the past few months and she had no intention to stop or reduce her training.

#### Life Domains

The interview was centred around well-being in eight life domains: health (physical), environment, intellectual growth, occupation, health (emotional), social life, financial status, and spiritual life (Swarbrick, 2006). Initially she had to consider the momentary aspect of well-being in these domains and then she had to recall her well-being in these life domains before her intense involvement in body building (i.e., performing it as a sport and competing in it). The outcome measure was the *perceived difference* between her past and present, recorded as positive change, no change, or negative change. As illustrated in Table 1, Evelyn perceives her well-being better in six out of eight life domains at present in contrast to the past when exercise was not as important as today. The fact that work and exercise take a tool on her social life is manifested as a partially negative change compared to the past, while no major change could be detected in her spiritual life. This trend of improvement after her intense involvement in exercise is also justified by her answer to a sort of summary question asking her to show on a ruler where is her overall well-being now and where was it before her dedication to body building; the current rating corresponded to a 7-8 on 10 partition while the rating of her past well-being was only between 3-4. Clearly, if one considers the middle point (5) as the separation between negative and positive appraisal, it might be assumed that the intensified involvement in sport triggered positive changes in Evelyn's well-being.

**Table 1:** Perceived Well-Being in Eight Life Domains

Life domain	At the time of interview	Before the heavy involvement in exercise training
Emotional	1. Appears balanced, self-confident, aware of the situation.	**1. Experienced emotional distress, due to family, financial, and relationship issues; had to seek professional help.**
Physical	2. Perceives to be in good physical health.	**2. Experienced stomach and cardiac problems.**
Environmental	3. Lives independently, has control over her life.	**3. Lived with a parent, was dependent on others.**
Occupational	4. Works in a full-time job.	**4. Studied, had no or part time job only.**
Intellectual	5. Faces work-related intellectual challenge at work.	**5. Faced challenges related to student life and studies.**
Financial	6. The financial situation is good, but it is not yet ideal.	**6. It was very unsatisfactory, had major financial hardship.**
Social	**7. It is not satisfactory, because work and training leaves no time for socialization, or for developing a lasting relationship.**	7. It was slightly better than now, but still not fully satisfactory.
Spiritual	8. No change in this domain; she is a believer with some interest in Astrology.	8. It was not different before; she was a believer with some interest in Astrology.

*Note*. Currently perceived well-being in eight life domains (Swarbrick, 2006) as compared to the recalled well-being before Evelyn's intense involvement in body building. The latter is also a current appreciation of past events; Therefore, the important outcome measure is the current state of the mind or thought. In bold fonts are the areas perceived as negative by Evelyn.

## Discussion

Considering her quantitative responses, Evelyn could be classified as symptomatic based on the EAI and on the DSM-5 classification, as extrapolated from gambling to exercise, she would be diagnosed with disordered exercise behaviour. The diagnosis would be justified by the nearly maximum score on obsessive passion (refer to Figure 1). However, her data on deprivation sensations are far from the maximum, she scored relatively high on harmonious passion and reported nearly maximum scores on commitment to exercise. At first glance, these figures appear to be controversial, but they are not as it should become clear after a deeper evaluation.

### Exercise Addiction Inventory (EAI): The Scoring Compared With the Interview

The EAI was built upon six symptoms commonly observed in all addictive behaviours (Terry et al., 2004). Salience, or high priority and preoccupation with exercise is the first of the six symptoms. Evelyn scored five, which is the maximum when completing the scale and she also reaffirmed during the interview that basically her life revolves around her sport. On "withdrawal symptoms" again she gave a maximum rating and during the interview she confirmed the ever presence of these symptoms and even listed some of its forms, which were guilt, insomnia, and nervousness. However, on the Deprivation Sensation Scale, Evelyn scored 44 out of 63 possible, which is just slightly over than two thirds maximal score, yet it is higher than that reported earlier (30) for body builders (Szabo et al., 1996). This issue will be treated separately below, not only because of an apparent discrepancy between the EAI score plus interview and the Deprivation Sensation Scale results, but also because early research relied solely on the presence of withdrawal symptoms in establishing the existence of exercise addiction (Szabo, Griffiths, & Demetrovics, 2016). Evelyn scored four, representing agreement, on the "mood modification" item of the EAI and she justified her answer by stating that she often uses exercise in dealing with life-stress. On "conflict" her scale and interview scores were dissonant, because on the former she gave a score of two, reflecting the rather disagree answer, but during the interview she claimed that she has experienced some conflict at work. This discrepancy was followed up during the interview and it turned out that when she was rating the questionnaire she focused on her close relationships only. On the final two symptoms gauging "tolerance" (rated as 1, which means strongly disagree) and on "relapse" (rated as 3, meaning neither agrees nor disagrees) the scale results were consistent with the interview. When these questions were further probed during the interview, Evelyn explained that she has a stable training plan for a while that needs no adjustment in volume. Concerning relapse, she explained her rating through the fact that since she became heavily involved in her sport, she never tried to cut down the amount of training that she performs, so she has perceived this item as not applicable to her sport and thus could neither agree or disagree.

The study shows that in the current case study the quantitative assessment of the EAI was mostly in accord with the responses gained during the interview. However, the misinterpretation of the conflict item led to a lower score on the EAI. Nevertheless, even if she would have scored 5 (maximum) on this item, she would have had a total score of 23 that is still not reaching the cut-off score of 24, which draws the line between *symptomatic* and *at-risk* exercise behaviour (Terry et al., 2004). Thus, according to the EAI score, Evelyn could be classified as symptomatic, but the EAI items (which reflect the common symptoms of addictions based on the Components Model [Griffiths, 2005]) have a justifiably different interpretation in Evelyn' and perhaps other athletes’ cases (Szabo et al., 2015). Indeed, salience is an expectable characteristic of the athletes trying to get higher on the competition ladder. Withdrawal symptoms are “normal reactions” to all activities that one adopts as a part of the daily life; in fact, bowlers also have experienced deprivation feelings at the times when they could not bowl, even though their symptoms were milder than those reported by body builders (Szabo et al., 1996). Conflict has the lowest factor loading on the EAI (Lichtenstein, Christiansen, Bilenberg, & Støving, 2014), but its presence is also “normal” in the highly committed athletes' life, because the training demands and associated obligations could result in interpersonal conflict (Szabo et al., 2015). The item “mood modification” in Evelyn's case (as complemented by the interview) reflects her coping with life stress. While this item could be related to good performance, appearance, and exercise-induced feeling states in Evelyn's case it emerges as a *coincidental* coping mechanism. I stress the term “coincidental”, because her motive for participation in sport is not to cope with stress *per se*, but to develop into a successful athlete. At the same time, she realizes that sport helps her in dealing with stress and she simply profits from the opportunity. While tolerance and relapse also have different meaning for exercise addicts and elite competing athletes (Szabo et al., 2015), these symptoms were not relevant in Evelyn's case. Summing up the EAI-based findings for Evelyn, the paper and pencil tool emerged to be in accord with the interview except for one item. The EAI would classify Evelyn as symptomatic for the risk of exercise addiction, and although four symptoms' presence out of six could be backed up with the results of the interview, Evelyn showed no signs of maladaptive exercise behaviour. She did not lose control over her training, even though occasionally she felt like doing a little surplus exercise above her usual training programme. Apart from a cut back in her social life, and her last university term studies suffering because of her training, she had no major losses in her life that could be attributed to her training. Thus, it can be argued that Evelyn *shows no signs of exercise addiction*, even though her quantitative (questionnaire) data would classify her as symptomatic.

### Making Sense of the Withdrawal Symptoms

**Figure 2:**
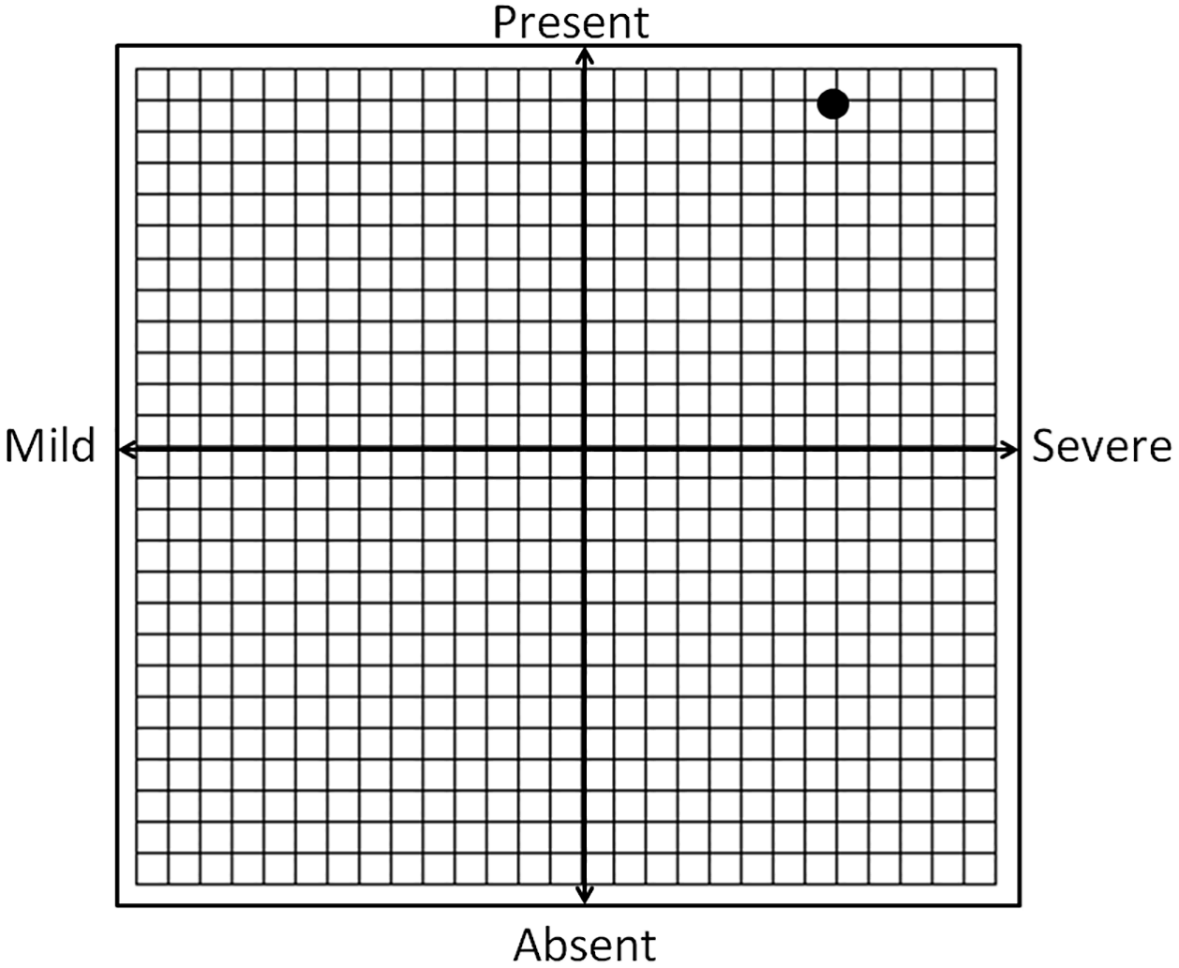
The conceptual link between the presence and the severity of withdrawal symptoms in exercise addiction, indicating the approximate position of Evelyn's scores with the black dot.

Evelyn scored the maximum (5) on the EAI withdrawal symptoms item and during the interview she also admitted experiencing unpleasant symptoms for the times when training is not possible. However, she only scored 69.8% of the maximum score on the Deprivation Sensations Scale. The slightly confounding results may be blamed on the tool's or response's reliability; but that would be a hazardous option. The fact is that the EAI is an *agreement-disagreement* scale on which the maximum score reflects a total agreement with the statement (i.e., that the respondent experiences withdrawal symptoms when exercise is not possible). This rating was corroborated by her responses during the interview. So why does it appear to be in discord with the deprivation score obtained on the Deprivation Sensation Scale? The answer is that the latter is a *frequency* scale on which the frequency of experiencing the symptoms yields a measure of the severity of the deprivation feelings. For example, if *all* symptoms are always *present* then they are quite severe compared to when only 5 out of 9 are being present with moderate frequency. Now this point may seem farfetched, but it is an important point that is being overlooked in the extant literature. As Szabo et al. (2016) have clarified, the mere presence of withdrawal symptoms is not indicative of exercise addiction, but their severity might be (Figure 2). In Evelyn's case the actual presence of the withdrawal symptoms for the times when she must miss a training session were confirmed, but they were not maximal in severity (refer to Figure 2), which explains the discord between the results of the EAI, backed up with the interview, and her ratings of the deprivation sensations.

### Obsessive Passion and Harmonious Passion

Evelyn scored a close to maximum score on obsessive passion (refer to Figure 1). Given the reported relationship between exercise addiction and obsessive passion (De La Vega et al., 2016; Schipfer & Stoll, 2015; Stenseng et al., 2011), her score may be an index of maladaptive, controlled exercise, characterized by a rigid involvement (Stenseng et al., 2011; Vallerand et al., 2003; Vallerand et al., 2007; Vallerand & Miquelon, 2007). However, competitive athletes seem to score higher on obsessive passion as well as harmonious passion than recreational exercisers (De La Vega et al., 2016). Evelyn's score on harmonious passion was lower (33) than on obsessive passion (40), but it was higher than the mean score of the recreational exercisers (29.1) in De La Vega et al.'s. (2016) study, while it was fitting the mean values reported for competitive athletes (33.3 - 33.9) in that work. These results make sense if one thinks about the controlling and rigid aspects of competitive sports; these characteristics stem from the discipline to follow a strict and demanding training regimen that, for the sake of success, virtually controls the athletes' lives. The high harmonious passion reflects the autonomously internalized activity, pride in success, and the joy of increasingly better performance. Thus, competitive athletes could be expected to exhibit high scores on both obsessive- and harmonious passion, one reflecting a surrender to conformity (e.g., “*I must do it to ..., I must do an extra session to..., Unless I push myself, I will not...*”, and so on). These self- and coach-*imposed* pressures are not mere choices, but perceived obligations to which the athlete needs to succumb to succeed. They are controlled internalizations that could stem from both intra- and interpersonal pressures (Vallerand, 2012). Evelyn justified the cases of the extra training sessions with feelings of guilt when she missed a training session and with perceived lack of achieving her goals in time, or a sort of dissatisfaction with her progress, which mirrors her intrapersonal pressure.

**Figure 3:**
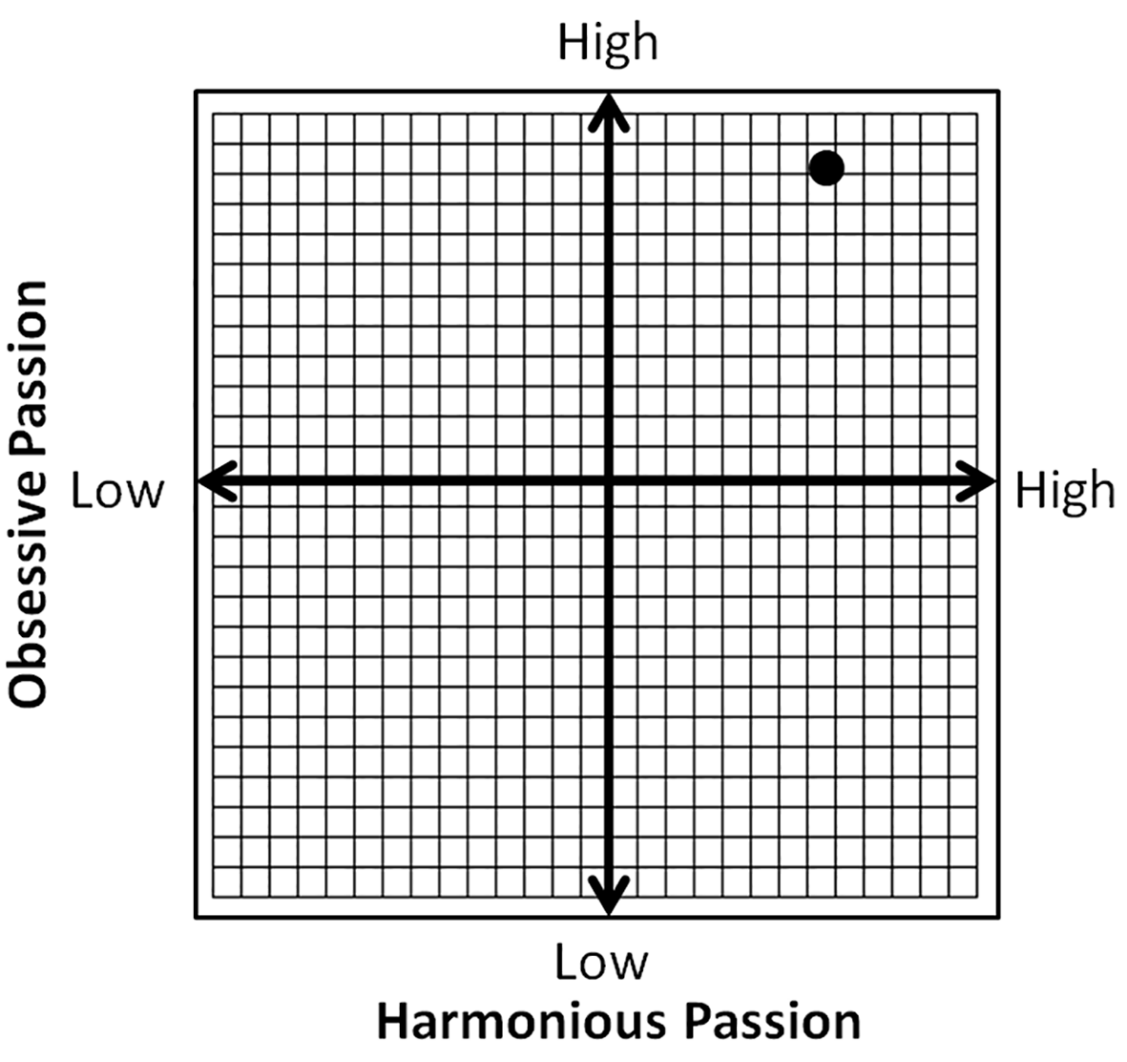
The conceptual association between obsessive- and harmonious passion, indicating the approximate position of Evelyn's scores with the black dot.

On the other hand, high levels of harmonious passion reflect the *chosen* aspects related to sport behaviour. They could involve the dedication, aspiration, and strong commitment mediated through the positive reinforcement of the regular training, stemming from an internal motivation^i^. However, it is more than internal motivation, because the dedicated athlete identifies herself with what she is doing (Vallerand, 2012), that is part of her identity. The greater this part is, the higher the passion scores will be; obsessive passion surfaces with conformity and self-imposed pressure to achieve her goal, while harmonious passion rises with pride, enjoyment, and satisfaction. Her scores on these scales (Figure 3), in accord with De La Vega et al.'s. (2016) findings, support the conjecture that there is a two-dimensional nature of passion in sport.

### Non-Substance Related Disorders Category of the DSM-5

Evelyn scored six out of nine of the symptoms enlisted for exercise disorder under the Non-Substance Related Disorders category of the DSM-5; scoring four or more on this category is an index of disordered behaviour (American Psychiatric Association, 2013). While adopting the symptoms of gambling disorder to exercise addiction could be criticized, there appears to be a valid ground for the practice (Potenza, 2014). The Exercise Dependence Scale (EDS; Hausenblas & Downs, 2002) was conceptualized based on the DSM-4 criteria for substance dependence. The problem is not with the drawn analogy between the two behavioural addictions, because analogy also exists between behavioural- and substance-related addictions (Alavi et al., 2012), but with the interpretation of these symptoms by elite athletes when they are presented for the evaluation of their training (see Table 2). Evelyn only denied three out of the nine DSM criteria: a) Item 3: trying to cut down on exercise; b) Item 7: lying about her exercise; and c) Item 9: relying on others to do things for her. All the other six criteria can be interpreted as part of who she is, or being an elite athlete. They are no longer *pathological* symptoms, but normal aspects of her training, whereas for a recreational exerciser may not be as “normal”. Indeed, Evelyn thought that those criteria suited her exercise behaviour. Examining the right column in Table 2, perhaps most readers would agree that those criteria, which could be symptoms for recreational exercisers, are *natural aspects* of the elite athletes' lives. Same with the EAI, which was developed with recreational exercisers, athletes usually show very high scores because the items have a *different meaning* to them (Szabo et al., 2015). Therefore, a great deal of confusion exists in the literature, unless high level of athletic involvement is paired with psychopathology. Indeed, Evelyn's appraisal of the DSM-5 criteria reflect the subjective appraisal of her athletic life as a successful body builder who longs for much more, and clearly *does not* reflect any sort of maladjustment.

**Table 2:** Adaptation of the DSM-5 Criteria for Gambling Disorder to Exercise Addiction and the Possible Interpretation of Each Criterion by the Elite Athlete (Last Column in Bold Letters)

DSM-5 Gambling Disorder criteria	As it applies to Exercise Addiction	What the symptom-criteria mirror in case of elite athletes
1. Need to gamble with increasing amount of money to achieve the desired excitement.	1. Needs to exercise more to achieve the same satisfaction as before.	**1. Needs to exercise to reach higher goals.**
2. Restless or irritable when trying to cut down or stop gambling.	2. Restless or irritable when must cut down or stop exercising.	**2. Hard feelings when losing training for an unwanted or unexpected reason.**
3. Repeated unsuccessful efforts to control, cut back on or stop gambling.	3. Repeated unsuccessful efforts to control, cut back on exercise.	**3. Does not normally want to cut down on training (unless injured).**
4. Frequent thoughts about gambling (such as reliving past gambling experiences, planning the next gambling venture, thinking of ways to get money to gamble).	4. Frequent thoughts about exercise (such as reliving past experiences, planning the next exercise session, thinking of ways to get more exercise).	**4. A normal salience for the elite athlete who must plan and evaluate workouts and schedule the days around the training.**
5. Often gambling when feeling distressed.	5. Often exercising when feeling distressed.	**5. May rely on exercise or training to deal with stress.**
6. After losing money gambling, often returning to get even (referred to as “chasing” one’s losses).	6. After insufficient or missed exercise, pushes hard to make up for it.	**6. Emerges as conformity, self-imposed pressure, obsessive passion; part of elite athletes' training.**
7. Lying to conceal gambling activity.	7. Lying to conceal problematic exercise behaviour.	**7. It s not applicable in most cases of elite athletes.**
8. Jeopardizing or losing a significant relationship, job or educational/career opportunity because of gambling.	8. Jeopardizing or losing a significant relationship, job or educational/career opportunity because of exercise.	**8. Being an elite athlete takes sacrifices.**
9. Relying on others to help with money problems caused by gambling.	9. Relying on others to help with issues, neglected chores, or obligations caused by exaggerated exercise.	**9. Due to the demand of training, time constraints, travel to competitions, may rely on others' help.**

### Commitment to Exercise

Evelyn scored nearly maximum points commitment measured with the '*passion criteria'* subscale of the Passion Scale (Figure 1) that reflects self-reported time-investment, affiliation, importance, involvement, and identity in the context of the athletic activity (De La Vega et al., 2016; Marsh et al., 2013). Although, interpreted as a positive feature of exercise behaviour, early research went so far to interpret high scores of commitment as addiction (Szabo, 2010). A high score of commitment reflects how hard a person wants to achieve a goal and the extent to which she is willing to make sacrifices (Locke, 1996). High level of commitment cannot be separated from obsessive passion, which embodies the internal pressure to do something, or to engage in an activity (Forest, Mageau, Sarrazin, & Morin, 2011). Evelyn's high scores on both commitment to exercise and obsessive passion may appear controversial at the first glance, but they are not, since a nearly maximal score of commitment reflects one's willingness to push herself to the extremes, and thus apply strong *internal pressure* in working towards a desired goal.

### Well-Being in Eight Life Domains Now and Before the Transition From Exercise to Sport

Out of the eight life domains of well-being (Swarbrick, 2006), Evelyn reported noticing improvements on six. Thus, although there was no change in her spiritual life and she suffered losses in social relationship, most areas of well-being have been improved, showing that her heavy involvement in exercise, now a sport, brought primarily positive changes in her life. Nevertheless, she experiences a major void in her life due to dissatisfaction with her social life. She sees this problem as an ongoing and stress-causing issue, because her work, training, and personal care do not leave sufficient time for socialization. She perceives this negative aspect in her well-being as a sacrifice for her dream-goals to become a successful and sponsored athlete and eventually blend her professional life with her sport. The sacrifice is in concordance with her high obsessive passion scores reflecting the self-imposed pressure to comply with training even at the expense of a much-desired need in another life domain. While unsatisfactory social life may be a negative experience due to exercise, it is not a form of loss, or ill consequence, that is reported in most case studies of exercise addiction (i.e., Schreiber & Hausenblas, 2015). While these results suggest that Evelyn feels much better in most life domains today than in the past, it should be appreciated that the latter appraisal of the events may be subject to memory distortion.

### Synopsis

While many people like Evelyn may claim that they are addicted to exercise (Szabo, 2010), they refer to their *high commitment* to training. The latter, as noted in Evelyn's case, is paired with high obsessive- and harmonious passion, one reflecting the self-imposed pressure while the other the benefits of training. Therefore, in accord with Evelyn's results, it is fair to anticipate high obsessive- and harmonious passion coupled with high level of commitment in successful athletes. *Together, they are healthy adaptations in striving towards a noble life-goal.* Evelyn is not addicted to exercise, even though she thought so, or may appear to be so based on the relatively high quantitative measures, as confirmed via the interview, which is a contradiction that supports Szabo et al.'s (2015) view that athletes interpret the symptoms of exercise addiction differently than recreational or leisure exercisers. The different interpretations gave rise to a false alarm in the literature suggesting that numerous athletes are prone to exercise addiction (McNamara & McCabe, 2012), often more prone than nonathletes (Blaydon & Lindner, 2002; Szabo et al., 2013a). Evelyn's proneness to exercise addiction, paired with no notable maladjustments in life, suggests that proneness (or risk determined with exercise addiction questionnaires) alone is *not* an index of dysfunction. Future studies should compare the differences in the levels of exercise addiction between non-competing recreational and competitive athletes, not simply on the bases of questionnaires, but by using follow-up interviews in the cases of established proneness or exercise addiction.

While Evelyn experiences deprivation sensations when she must miss her training, her scores reflecting the severity of the symptoms, in terms of type and frequency, were lower, which supports earlier contentions that the presence of withdrawal symptoms, or deprivation sensation, does not pair up with exercise addiction, but their severity does (Szabo, 2010). Finally, in the period following her intense involvement in exercise, turning into sport, Evelyn had experienced positive changes in most life areas, which underpins the conclusion of the study that she has a passion for, but not addiction to exercise. These results suggest that actual cases of exercises addiction may be occur less frequently than expected based on the risk assessment (using a nomothetic/ questionnaire approach) in the literature.

### Limitations

The study is not without limitation. The lack of generalizability is one obvious limitation. Lack of random selection (which is impossible in this area) and the participant's knowledge about the topic of the interview could have biased her responses. Like in most case studies, the author must rely on the inferred honesty of the participant. Further, the exercise *per se* and competition aspect of Evelyn's sport cannot be untangled, but so is that of competitive athletes studied in dozens of nomothetic investigations. Perhaps the explanation of the difference in the prevalence of exercise addiction, attributed to different interpretation of exercise addiction items by recreational and competing athletes (Szabo et al., 2015) is an issue of an additional variable (i.e., competition) in the latter group. This feature should be systematically investigated in future works by a direct comparison of two matched groups, one recreational and one competitive. Still another limitation, is in comparing the eight life domains (Swarbrick, 2006) at present and in the past, since the latter relied on retrospective answers that could be subject to memory distortion. Perhaps only longitudinal studies could overcome this limitation.

### Conclusions

The presented case study provides a real-life example of how *self-perceived* exercise addiction is only a mere reflection of both obsessive and harmonious passion in the maximal commitment to a sporting activity in a female body builder. The case study supports previous contentions that not the presence, but the strength of the deprivation sensations associated with exercise addiction may be instrumental in separating healthy- from unhealthy exercise pattern. The parallel between very high scores of obsessive passion and commitment in the examined person suggest that high internal pressure related to conformity in the training regimen may be part of the high commitment and that high obsessive passion can co-occur with high harmonious passion, the latter reflecting the joy and satisfaction derived from the results of training. The case study also revealed that the DSM-5 criteria for Non-Substance Related Disorders, when adopted for disordered exercise behaviour, may not apply, because of valid *alternative interpretation* of the nine criteria by the highly committed athlete; the lack of concordance of the DSM-5 outcome and the scores on the Exercise Addiction Inventory further justifies this point. The message from this case study is that future studies should look at passion and commitment too while examining the risk for exercise addiction, and more importantly that questionnaire-based risk scores may not reflect actual risk for dysfunction, thus their conceptual internal validity needs to be revisited.

### Take Home Message

It is possible that nomothetic (quantitative) research, assessing the *risk* for exercise addiction, embeds a confounding overlap between passion, commitment, and exercise addiction, which yields false estimate of the actual risk and prevalence of the dysfunction. Despite self-perceived exercise addiction, confirmed by various measures used in nomothetic research and DSM criteria for addiction, no signs of dysfunction and/or psychopathology could be detected in a female body builder, who is a competing athlete. The bulk of past research on exercise addictions should be re-evaluated with a view on the unlikely problematic behaviour mirrored by questionnaire scores.

## References

[r1] Alavi S. S. Ferdosi M. Jannatifard F. Eslami M. Alaghemandan H. Setare M. (2012). Behavioral addiction versus substance addiction: Correspondence of psychiatric and psychological views. International Journal of Preventive Medicine, 3(4), 290–294. 22624087 PMC3354400

[r2] Allegre B. Therme P. Griffiths M. (2007). Individual factors and the context of physical activity in exercise dependence: A prospective study of ‘ultra-marathoners’. International Journal of Mental Health and Addiction, 5(3), 233–243. doi:. 10.1007/s11469-007-9081-9

[r3] American Psychiatric Association. (2013). *Diagnostic and statistical manual of mental disorders* (5th ed). Arlington, VA, USA: American Psychiatric Publishing.

[r4] Back, J. (2016). *Profiles of exercise dependence–A person centred approach to study potential mechanisms* (Master's thesis, Academy for Health and Welfare, Halmstad University, Sweden). Retrieved from http://www.diva-portal.org/smash/get/diva2:940447/FULLTEXT02

[r5] Blaydon M. J. Lindner K. J. (2002). Eating disorders and exercise dependence in triathletes. Eating Disorders, 10(1), 49–60. doi:. 10.1080/106402602753573559 16864244

[r6] Brewer J. A. Potenza M. N. (2008). The neurobiology and genetics of impulse control disorders: Relationships to drug addictions. Biochemical Pharmacology, 75(1), 63–75. doi:. 10.1016/j.bcp.2007.06.043 17719013 PMC2222549

[r7] Caspersen C. J. Powell K. E. Christenson G. M. (1985). Physical activity, exercise, and physical fitness: Definitions and distinctions for health-related research. Public Health Reports, 100(2), 126–131. Retrieved from https://www.ncbi.nlm.nih.gov/pmc/articles/PMC1424733/pdf/pubhealthrep00100-0016.pdf. 3920711 PMC1424733

[r8] De La Vega R. Parastatidou I. S. Ruíz-Barquín R. Szabo A. (2016). Exercise addiction in athletes and leisure exercisers: The moderating role of passion. Journal of Behavioral Addictions, 5(2), 325–331. doi:. 10.1556/2006.5.2016.043 27363466 PMC5387784

[r9] Doggett, R., & Koegel, R. L. (2013). Negative reinforcement. In F. R. Volkmar (Ed.), *Encyclopedia of autism spectrum disorders.* New York, NY, USA: Springer.

[r10] Egorov A. Y. Szabo A. (2013). The exercise paradox: An interactional model for a clearer conceptualization of exercise addiction. Journal of Behavioral Addictions, 2(4), 199–208. doi:. 10.1556/JBA.2.2013.4.2 25215201 PMC4154576

[r11] Forest J. Mageau G. A. Sarrazin C. Morin E. M. (2011). “Work is my passion”: The different affective, behavioural, and cognitive consequences of harmonious and obsessive passion toward work. Canadian Journal of Administrative Sciences / Revue Canadienne Des Sciences de l’Administration*,* 28(1), 27–40. doi: 10.1002/cjas.170

[r12] Foulds H. J. A. Bredin S. S. D. Charlesworth S. A. Ivey A. C. Warburton D. E. R. (2014). Exercise volume and intensity: A dose–response relationship with health benefits. European Journal of Applied Physiology, 114(8), 1563–1571. doi:. 10.1007/s00421-014-2887-9 24770699

[r13] Goodman A. (1990). Addiction: Definition and implications. British Journal of Addiction, 85(11), 1403–1408. doi:. 10.1111/j.1360-0443.1990.tb01620.x 2285834

[r14] Griffiths M. D. (1996). Behavioural addiction: An issue for everybody? Employee Counselling Today, 8(3), 19–25. doi:. 10.1108/13665629610116872

[r15] Griffiths M. D. (1997). Exercise addiction: A case study. Addiction Research, 5(2), 161–168. doi:. 10.3109/16066359709005257

[r16] Griffiths M. D. (2005). A ‘components’ model of addiction within a biopsychosocial framework. Journal of Substance Use, 10(4), 191–197. doi:. 10.1080/14659890500114359

[r17] Griffiths M. D. Szabo A. Terry A. (2005). The Exercise Addiction Inventory: A quick and easy screening tool for health practitioners. British Journal of Sports Medicine, 39(6), e30. 10.1136/bjsm.2004.017020 15911594 PMC1725234

[r18] Griffiths M. D. Urbán R. Demetrovics Z. Lichtenstein M. B. de la Vega R. Kun B. Szabo A. (2015). A cross-cultural re-evaluation of the Exercise Addiction Inventory (EAI) in five countries. Sports Medicine - Open, 1(1), 5. 10.1186/s40798-014-0005-5 27747842 PMC4532705

[r19] Hausenblas H. A. Downs D. S. (2002). How much is too much? The development and validation of the Exercise Dependence Scale. Psychology & Health, 17(4), 387–404. doi:. 10.1080/0887044022000004894

[r20] Hausenblas H. A. Schreiber K. Smoliga J. M. (2017). Addiction to exercise. BMJ*,* 357, j1745. 10.1136/bmj.j1745 28446435

[r21] Kotbagi G. Muller I. Romo L. Kern L. (2014). Pratique problématique d’exercice physique: Un cas clinique. Annales Médico-Psychologiques, Revue Psychiatrique, 172(10), 883–887. 10.1016/j.amp.2014.10.011

[r22] Kovacsik R. Griffiths M. D. Pontes H. M. Soós I. de la Vega R. Ruíz-Barquín R. Szabo A (2018). The role of passion in exercise addiction, exercise volume, and exercise intensity in long-term exercisers. International Journal of Mental Health and Addiction. Advance online publication 10.1007/s11469-018-9880-1

[r23] Lichtenstein M. B. Christiansen E. Bilenberg N. Støving R. K. (2014). Validation of the exercise addiction inventory in a Danish sport context. Scandinavian Journal of Medicine & Science in Sports, 24(2), 447–453. 10.1111/j.1600-0838.2012.01515.x 22882175

[r24] Locke E. A. (1996). Motivation through conscious goal setting. Applied & Preventive Psychology, 5(2), 117–124. doi:. 10.1016/S0962-1849(96)80005-9

[r25] Marsh H. W. Vallerand R. J. Lafrenière M.-A. K. Parker P. Morin A. J. S. Carbonneau N. Paquet Y. (2013). Passion: Does one scale fit all? Construct validity of two-factor Passion Scale and psychometric invariance over different activities and languages. Psychological Assessment, 25(3), 796–809. doi:. 10.1037/a0032573 23647035

[r26] McNamara J. McCabe M. P. (2012). Striving for success or addiction? Exercise dependence among elite Australian athletes. Journal of Sports Sciences, 30(8), 755–766. doi:. 10.1080/02640414.2012.667879 22420455

[r27] Miele G. M. Tilly S. M. First M. Frances A. (1990). The definition of dependence and behavioural addictions. British Journal of Addiction, 85(11), 1421–1423. doi:. 10.1111/j.1360-0443.1990.tb01623.x 2285837

[r28] Mónok K. Berczik K. Urbán R. Szabo A. Griffiths M. D. Farkas J. Demetrovics Z. (2012). Psychometric properties and concurrent validity of two exercise addiction measures: A population wide study. Psychology of Sport and Exercise, 13(6), 739–746. doi:. 10.1016/j.psychsport.2012.06.003

[r29] Paradis K. F. Cooke L. M. Martin L. J. Hall C. R. (2013). Too much of a good thing? Examining the relationship between passion for exercise and exercise dependence. Psychology of Sport and Exercise, 14(4), 493–500. doi:. 10.1016/j.psychsport.2013.02.003

[r30] Parastatidou I. S. Doganis G. Theodorakis Y. Vlachopoulos S. P. (2012). Exercising with passion: Initial validation of the Passion Scale in exercise. Measurement in Physical Education and Exercise Science, 16(2), 119–134. doi:. 10.1080/1091367X.2012.657561

[r31] Parastatidou I. S. Doganis G. Theodorakis Y. Vlachopoulos S. P. (2014). The mediating role of passion in the relationship of exercise motivational regulations with exercise dependence symptoms. International Journal of Mental Health and Addiction, 12(4), 406–419. doi:. 10.1007/s11469-013-9466-x

[r32] Pasman L. J. Thompson J. K. (1988). Body image and eating disturbance in obligatory runners, obligatory weightlifters, and sedentary individuals. International Journal of Eating Disorders, 7(6), 759–769. doi:. 10.1002/1098-108X(198811)7:6<759::AID-EAT2260070605>3.0.CO;2-G

[r33] Potenza M. N. (2014). Non-substance addictive behaviors in the context of DSM-5. Addictive Behaviors, 39(1), 1–2. doi: 10.1016/j.addbeh.2013.09.004 24119712 PMC3858502

[r34] Robbins J. M. Joseph P. (1985). Experiencing exercise withdrawal: Possible consequences of therapeutic and mastery running. Journal of Sport Psychology, 7(1), 23–39. doi:. 10.1123/jsp.7.1.23

[r35] Rousseau F. L. Vallerand R. J. Ratelle C. F. Mageau G. A. Provencher P. J. (2002). Passion and gambling: On the validation of the Gambling Passion Scale (GPS)*.* Journal of Gambling Studies, 18(1), 45–66. 10.1023/A:1014532229487 12050847

[r36] Schipfer M. Stoll O. (2015). OR-77: Exercise-Addiction/Exercise-Commitment-Model (EACOM). Paper presented at the 14th European Congress of Sport Psychology, Bern, Switzerland. Journal of Behavioral Addictions, 4(S1), 35–37.26014672

[r37] Schreiber, K., & Hausenblas, H. A. (2015). *The truth about exercise addiction: Understanding the dark side of thinspiration.* Lanham, MD, USA: Rowman & Littlefield.

[r38] Sicilia Á. Alcaraz-Ibáñez M. Lirola M.-J. Burgueño R. (2017). Influence of goal contents on exercise addiction: Analysing the mediating effect of passion for exercise. Journal of Human Kinetics, 59(1), 143–153. 10.1515/hukin-2017-0154 29134055 PMC5680693

[r39] Stenseng F. Rise J. Kraft P. (2011). The dark side of leisure: Obsessive passion and its covariates and outcomes. Leisure Studies, 30(1), 49–62. doi:. 10.1080/02614361003716982

[r40] Swarbrick M. (2006). A wellness approach. Psychiatric Rehabilitation Journal, 29(4), 311–314. doi:. 10.2975/29.2006.311.314 16689042

[r41] Szabo, A. (2000). Physical activity as a source of psychological dysfunction. In S. J. Biddle, K. R. Fox, & S. H. Boutcher (Eds.), *Physical activity and psychological well-being* (pp. 130-153). London, United Kingdom: Routledge.

[r42] Szabo, A. (2010). *Addiction to exercise: A symptom or a disorder*? Hauppauge, NY, USA: Nova Science.

[r43] Szabo A. De La Vega R. Ruiz-Barquín R. Rivera O. (2013 a). Exercise addiction in Spanish athletes: Investigation of the roles of gender, social context and level of involvement. Journal of Behavioral Addictions, 2(4), 249–252. doi:. 10.1556/JBA.2.2013.4.9 25215208 PMC4154578

[r44] Szabo A. Frenkl R. Caputo A. (1996). Deprivation feelings, anxiety, and commitment in various forms of physical activity: A cross-sectional study on the internet. Psychologia, 39, 223–230.

[r45] Szabo A. Griffiths M. D. (2007). Exercise addiction in British sport science students. International Journal of Mental Health and Addiction, 5(1), 25–28. doi:. 10.1007/s11469-006-9050-8

[r46] Szabo A. Griffiths M. D. De La Vega R. M. Mervó B. Demetrovics Z. (2015). Methodological and conceptual limitations in exercise addiction research. The Yale Journal of Biology and Medicine, 88(3), 303–308. 26339214 PMC4553651

[r47] Szabo, A., Griffiths, M. D., & Demetrovics, Z. (2013b). Psychology and exercise. In D. Bagchi, S. Nair, & C. K. Sen (Eds.), *Nutrition and enhanced sports performance* (pp. 65-73). New York, NY, USA: Academic Press. 10.1016/b978-0-12-396454-0.00006-0

[r48] Szabo, A., Griffiths, M. D., & Demetrovics, Z. (2016). Exercise addiction. In V. R. Preedy (Ed.), *Neuropathology of Drug Addictions and Substance Misuse: Volume 3. General processes and mechanisms, prescription medications, Caffeine and Areca, polydrug misuse, emerging addictions and non-drug addictions* (pp. 984-992). 10.1016/b978-0-12-800634-4.00097-4

[r49] Terry A. Szabo A. Griffiths M. (2004). The exercise addiction inventory: A new brief screening tool. Addiction Research and Theory, 12, 489–499. doi:. 10.1080/16066350310001637363

[r50] Tóth-Király I. Bõthe B. Rigó A. Orosz G. (2017). An illustration of the Exploratory Structural Equation Modeling (ESEM) framework on the Passion Scale. Frontiers in Psychology, 8, 1968. 10.3389/fpsyg.2017.01968 29163325 PMC5681952

[r51] Vallerand R. J. (2010). On passion for life activities: The dualistic model of passion. Advances in Experimental Social Psychology, 42, 97–193. 10.1016/S0065-2601(10)42003-1

[r52] Vallerand R. J. (2012). From motivation to passion: In search of the motivational processes involved in a meaningful life. Canadian Psychology, 53(1), 42–52. doi:. 10.1037/a0026377

[r53] Vallerand R. J. Blanchard C. Mageau G. A. Koestner R. Ratelle C. Leonard M. Marsolais J. (2003). Les passions de l’ame: On obsessive and harmonious passion. Journal of Personality and Social Psychology, 85(4), 756–767. doi:. 10.1037/0022-3514.85.4.756 14561128

[r54] Vallerand, R. J., & Miquelon, P. (2007). Passion for sport in athletes. In D. Lavallee & S. Jowett (Eds.), *Social psychology in sport* (pp. 249-262). Champaign, IL, USA: Human Kinetics.

[r55] Vallerand R. J. Rousseau F. L. Grouzet F. M. E. Dumais A. Grenier S. Blanchard C. M. (2006). Passion in sport: A look at determinants and affective experiences. Journal of Sport & Exercise Psychology, 28(4), 454–478. 10.1123/jsep.28.4.454

[r56] Vallerand R. J. Salvy S.-J. Mageau G. A. Elliot A. J. Denis P. Grouzet F. M. E. Blanchard C. (2007). On the role of passion in performance. Journal of Personality, 75(3), 505–534. doi:. 10.1111/j.1467-6494.2007.00447.x 17489890

[r57] Veale, D. (1995). Does primary exercise dependence really exist? In J. Annett, B. Cripps, & H. Steinberg (Eds.), *Exercise addiction: Motivation for participation in sport and exercise* (pp. 1-5). Leicester, United Kingdom: British Psychological Society.

